# Kissing and the Bible

**Published:** 1892-06

**Authors:** 


### KISSING AND THE BIBLE.

Kissing plays as important a part in the Bible as it does in modern civilization. The *Herald* says that when doctors of divinity hold that every word and every letter of the Scripture is inspired and is literal truth, the kisses recorded in the Scriptures comes forth to torment them, even as the Bostonian tormented the vigilant guardian of demure deportment in the thoroughfares. In the Book of Psalms ii., 11, Protestant version, we read: "Kiss the son lest he be angry." The Catholic version is "Embrace discipline, lest at any time the Lord be angry." Here is clearly a confusion of translations. Which is right? In the tenth chapter of First Samuel the anointment of Saul is recorded, and the kissing of him by Samuel is related in the two versions in identical words. Why did they differ in the first? It is not a matter of verbal scholarship? And since the difference is obvious and is irreconcilable by scholarship for lack of sufficient knowledge or precise equivalents in languages, what becomes of literal translations—at least in translations? As Saint Luke relates the betrayal of Christ by Judas, the Latin vulgate uses oscular for the verb to kiss and osculum for the noun. English versions concur in using the kiss. Yet the Greek contains nothing to justify it. The Greek word means primarily an expression of respectful love, an embrace, a deferential and loving salutation. Philology is the most modern of sciences. By it alone can these discrepancies in the versions of the Scriptures be reconciled and their conflicting aspects removed. Until philology has said the last word on verbal equivalents, is it not premature to claim transliteral inspiration, especially in texts of whose origin nothing is absolutely known?

